# The Therapeutic and Diagnostic Potential of Phospholipase C Zeta, Oocyte Activation, and Calcium in Treating Human Infertility

**DOI:** 10.3390/ph16030441

**Published:** 2023-03-15

**Authors:** Haia M. R. Abdulsamad, Zoha F. Murtaza, Hessa M. AlMuhairi, Wjdan S. Bafleh, Salma A. AlMansoori, Shaikha A. AlQubaisi, Hamdan Hamdan, Junaid Kashir

**Affiliations:** 1 Department of Physiology and Immunology, College of Medicine and Health Sciences, Khalifa University, Abu Dhabi 127788, United Arab Emirates; 2 Department of Biology, College of Arts and Science, Khalifa University, Abu Dhabi 127788, United Arab Emirates; 3 Department of Comparative Medicine, King Faisal Specialist Hospital and Research Centre, Riyadh 12713, Saudi Arabia

**Keywords:** phospholipase C zeta (PLCzeta), oocyte activation, male infertility, sperm, fertilisation, calcium

## Abstract

Oocyte activation, a fundamental event during mammalian fertilisation, is initiated by concerted intracellular patterns of calcium (Ca^2+^) release, termed Ca^2+^ oscillations, predominantly driven by testis-specific phospholipase C zeta (PLCζ). Ca^2+^ exerts a pivotal role in not just regulating oocyte activation and driving fertilisation, but also in influencing the quality of embryogenesis. In humans, a failure of Ca^2+^ release, or defects in related mechanisms, have been reported to result in infertility. Furthermore, mutations in the PLCζ gene and abnormalities in sperm PLCζ protein and RNA, have been strongly associated with forms of male infertility where oocyte activation is deficient. Concurrently, specific patterns and profiles of PLCζ in human sperm have been linked to parameters of semen quality, suggesting the potential for PLCζ as a powerful target for both therapeutics and diagnostics of human fertility. However, further to PLCζ and given the strong role played by Ca^2+^ in fertilisation, targets down- and up-stream of this process may also present a significantly similar level of promise. Herein, we systematically summarise recent advancements and controversies in the field to update expanding clinical associations between Ca^2+^-release, PLCζ, oocyte activation and human fertility. We discuss how such associations may potentially underlie defective embryogenesis and recurrent implantation failure following fertility treatments, alongside potential diagnostic and therapeutic avenues presented by oocyte activation for the diagnosis and treatment of human infertility.

## 1. Introduction

Fertilization is a multistep process that is initiated by the interaction of sperm and the layer surrounding the oocyte, or what is known as zona pellucida (ZP), after which the sperm and oolemma will interact. This results in a signal transduction cascade, which will convert the oocyte into a diploid zygote, through a series of collective processes termed oocyte activation (OA). OA involves well-defined morphological and biochemical endpoints that occur following sperm-oocyte interaction, such as the resumption of meiosis II, prevention of polyspermy, and cortical granule exocytosis. These endpoints can vary in duration; some will require minutes, and some will require hours after the interaction, but calcium levels are critical for all of them [1]. Prior to fertilisation, the oocyte first arrests in prophase I in the pre-ovulatory phase [2], until the release of luteinizing hormone (LH), which resumes meiosis until the metaphase II (MII) stage where another meiotic arrest occurs until fertilisation, regulated by cAMP. Increasing cAMP analogues or cAMP phosphodiesterase inhibitors will prevent oocyte maturation and will keep it arrested. G_s_ and G_s_-coupled receptors [GPR3 and GPR12] play a major role in meiotic arrest because the activation of G_s_ will increase the level of cAMP [3]. Numerous lines of investigation have established that the underlying factor of key importance at OA are intracellular Ca^2+^ ions [4].

Indeed, many experiments have demonstrated the importance of Ca^2+^ at OA in mammalian and non-mammalian species [5], with further studies suggesting that the specific profile of Ca^2+^ release at fertilisation can exert effects upon postnatal growth and the weight of mice offspring [6]. Intracellular Ca^2+^ release is a crucial component of OA, occurring in a dramatic wave-like manner, starting from the point of fusion to all over the oocyte. Generally, this increase in intracellular Ca^2+^ release seems mediated in an inositol 1,4,5-triphosphate (IP_3_) receptor-dependent manner and from Ca^2+^ stores, predominantly being the endoplasmic reticulum (ER). While in some species, such as Xenopus and sea urchins, there is a single wave of Ca^2+^ release, in others, such as mammals, this release pattern occurs in an oscillatory manner, the amplitude and frequency of which vary in duration, amplitude, and frequency depending on the species. Generally, Ca^2+^ oscillations are a linear result of IP_3_ activation [7]. Many experiments have observed IP_3_ peaks preceding Ca^2+^ release in oocytes during fertilisation [8], while other experiments also observed that Ca^2+^ ions could also initiate oocyte activation following microinjection in oocytes [8,9]. The down-regulation of IP_3_ receptors in hamster and mouse oocytes inhibited Ca^2+^ oscillations and oocyte activation [10,11,12,13]. 

Normally, basal cytosolic Ca^2+^ levels in the oocyte are kept relatively low compared to the extracellular environment, making the cytosol a favourable site for minor Ca^2+^ variation, as a response to extracellular or intracellular Ca^2+^ signalling [14]. The Ca^2+^ oscillation signals the start as a result of the disturbance of cytosolic Ca^2+^ equilibrium. This disturbance occurs as a response to stimulatory signals that act on Ca^2+^ receptors which are found either in the cytoplasmic membrane or ER (Ca^2^ store) and will result in an increase in cytoplasmic Ca^2+^ [15]. Coordinated calcium waves will be produced by internal Ca^2+^ stores, where a sudden increase of Ca^2+^ will induce more Ca^2+^ release through a series of events as positive feedback [16], a process termed calcium-induced calcium release (CICR). The most essential mediator for Ca^2+^ waves is inositol trisphosphate receptors (IP3Rs) which are found abundantly as calcium-releasing channels on ER in the cytosol [17]. IP_3_ resulting from various cascades produced by extracellular stimulants will bind to IP3Rs, causing a conformational change to Ca^2+^ channels found on the cell membrane leading to Ca^2+^ influx into the cell [18]. Following sperm penetration, Ca^2+^ oscillations are initiated that are critical for OA and the completion of meiosis II [19]. Ca^2+^ oscillations will activate Ca^2+^ calmodulin-dependent kinase II (CaMKII) which then will activate an anaphase-promoting complex (APC) [20]. The activation of the latter will then degrade securin and cyclin B1 (CCNB1) (cell cycle regulators) [21]. The degradation of such regulators enables cell cycle progression and segregation of sister chromatids, and thus (perhaps indirectly) may also control the occurrence of abnormal chromatid segregation and aneuploidy (for review see Jones and Lane [22]), although the exact mechanisms underlying this remain to be fully elucidated. Ca^2^ oscillation has also proven to play a major role in other developmental stages at the genome level and nuclear signalling level [19,21,23,24].

In mammalian oocytes, all of the events following Ca^2+^ oscillation occur in the temporal order, unlike non-mammalian cells where all of the events occur simultaneously after being exposed to single Ca^2+^ transients. Therefore, mammalian cells are more reactive to the frequency, duration, and amplitude of Ca^2+^ release. Along with the accuracy of the oscillations, the mature oocyte that is coordinated by important organelles, such as ER and mitochondria, are essential to maintain the periodical increase in Ca^2+^ [25].

## 2. Endoplasmic Reticulum (ER)

Ca^2+^ is pumped against a concentration gradient from the cytoplasm into the ER by the plasma membrane pump sarco-ER Ca^2+^-ATPase (SERCA). Three genes in mammals (ATP2A1–3) are responsible for producing three different isoforms of SERCA (SERCA1–3), and through alternative splicing, they produce 11 SERCA isoforms. Each one of these isoforms has a location, developmental expression, and most importantly, a unique affinity and sensitivity to Ca^2+^. Like any Ca^2+^ ATPase, SERCA’s functional structure is trans-membranal, containing three cytoplasmic domains (phosphorylation and nucleotide-binding domains in addition to the actuator) and ten membrane-spanning helices. The transmembrane domain contains two Ca^2+^-binding sites, making SERCA capable of transporting two Ca^2+^ ions per ATP. Any general plasma membrane ATPase inhibitor, such as orthovanadate, is able to inhibit an undetermined SERCA isoform or a specific one, such as thapsigargin [26].

Many studies have illustrated the influence of SERCA2 isoforms in sustaining Ca^2+^ oscillation during fertilisation in animal models, such as *Xenopus*, frogs, and mice. Interestingly, thapsigargin treatment significantly reduced the magnitude and duration of the first Ca^2+^ peak and oscillation persistence. During oocyte maturation, SERCA2B protein levels remain constant but are redistributed spatially from diffuse patterns to cortical clusters mimicking ER redistribution. This arrangement will allow SERCA to pump closer to IP3 receptors. This would be necessary for depletion that follows fertilisation, as it will facilitate the refilling of Ca^2+^ stores in ER [26].

## 3. Oocyte Mitochondria

Prior to oocyte and mitochondria maturation, the granulosa cells and cumulus provide the cell with energy. Following ovulation, the mitochondria start to activate and become the main source of energy in the mature oocyte [27]. Further to meeting the energy requirements of the oocyte (and subsequent embryo), the ATP supplied by the mitochondria also plays a critical role in genetic stability, due to its function in assembling microtubule spindles during meiosis [I and II]. Indeed, any decrease in ATP levels will cause chromosome rearrangements in the cells and that will lead to genetic disorders. Furthermore, the mitochondria is one of the major players in cellular homeostasis, in particular Ca^2+^ intracellular homeostasis. Alteration in cellular homeostasis depends on the change of Ca^2+^ concentration, for example, if the concentration of Ca^2+^ entering the mitochondria decreases more than it should, this will cause a bioenergy disaster. Moreover, if the concentration of Ca^2+^ in the cell increases, causing apoptosis because the abnormal Ca^2+^ concentration disrupts the oxidative phosphorylation and can open the transition pore in mitochondria, this will cause mitochondrial dysfunction [27].

## 4. Ca^2+^ Oscillation Models

Ca^2+^ oscillations have been proven for a long time in many studies as an important step of OA, but the exact mechanism that results in the oscillation, specifically in relation to gamete fusion, remains unclear. Few hypotheses have been suggested [28].

## 5. The Ca^2+^ Conduit Model

Based on the sea urchin model, it was suggested that the infusion of a considerable amount of Ca^2+^ into the oocyte right after sperm fusion would lead to Ca^2+^-induced Ca^2+^ release, allowing Ca^2+^-influx into the oocyte. However, this model was not successful on other animal models, such as mice and ascidians. Moreover, experiments emphasize the importance of the IP_3_ pathway to release and maintain calcium in OA [28].

## 6. The Membrane Receptor Model

The basic theory underlying this model suggests that OA would result from the interaction between a specific sperm-ligand and oocyte-receptor, activating a phospholipase C (PLC) inside the oocyte. However, such assertions were supported by indirect evidence and the experiments involved overexpressed G-protein linked receptors which might be responsible for activating PLC-β, as a response to gamete interactions and the corresponding application of ligands. Some experiments showed Ca^2+^ release by injecting the hydrolysis-resistant GTP analogue GTP-γS, in sea urchins and frog eggs. However, resultant patterns of Ca^2+^ release were not comparable to that at fertilisation, specifically in mammalian cells. Moreover, the direct injection of sperm into the oocyte cytosol using intracytoplasmic sperm injection (ICSI) can undergo successful fertilisation and embryogenesis, without any such membrane-membrane interactions [28], creating doubt regarding the veracity of this model, at least within mammals. Interestingly, ICSI can also yield Ca^2+^ oscillations and the production of considerable IP_3_ levels [29,30,31,32].

## 7. The Soluble Sperm Factor

This model suggests that a soluble sperm factor is released into the oocyte during or immediately after gamete fusion, which in turn is responsible for OA. Injection of sperm cytosolic extracts into the eggs/oocytes of sea urchins, mice, humans, pigs, and cows triggered the characteristic series of Ca^2+^ oscillations seen at fertilisation, while also producing the subsequent events of OA [33,34,35]. One would also expect that considering the IP_3_-mediated nature of Ca^2+^ release in mammalian oocytes, it would be suitable to consider that a phosphoinositide (PI)-specific PLC-associated pathway is simulated [32]. Indeed, the characteristic pattern of Ca^2+^ release at fertilisation is not stimulated by Ca^2+^ injection (although in suitably high concentrations, this can result in OA), nor does injection of IP_3_ or stimulating G-proteins (although these do result in an insufficient series of Ca^2+^ release highly different from those at fertilisation) [32,36]. Most scientific opinion suggests that the correct theory is indeed a specific soluble protein delivered to the oocyte by the sperm, resulting in Ca^2+^ release and OA. Indeed, given the specifications underlying the signalling mechanisms underlying OA, most opinions suggest a PLC-mediated mechanism is the essential factor to initiate the IP_3_ pathway for OA [28,32].

## 8. The Mammalian Sperm Factor: Phospholipase C Zeta

A number of factors and proteins have been proposed to be the sperm factor, including the post-acrosomal WW-domain binding protein (PAWP), where its implied function in OA is through a yes-associated protein (YAP) to activate PLC-γ, similar to what happens in *Xenopus* eggs [37,38]. The role of PAWP was seen when the binding of a competitive inhibitor to a PPGY peptide, which is derived from PAWP in murine and human oocytes, inhibited the rrelease of Ca^2+^ [38,39]. Microinjection of recombinant PAWP into mouse oocytes did not cause Ca^2+^ oscillations, while the suggested signalling pathway associated with PAWP seemingly has no relevance to OA [40]. A further candidate sperm factor has also included a truncated c-kit receptor, tr-kit, which was able to induce parthenogenetic mouse OA via phosphorylation and activation of PLCγ1 [41,42] (like the proposed action of PAWP). However, these findings have yet to be independently verified.

The series of Ca^2+^ oscillations that are seen in OA that are attributed to be the function of the “sperm factor” is believed to be the direct result of Ca^2+^ release via (IP_3_-mediated reactions [10,15,28,43,44,45], and PLCs are a class of enzymes well characterised to be involved in the catabolism of phosphatidylinositol 4,5-bisphosphate (PIP_2_) into IP_3_ and diacylglycerol (DAG) [28,46,47,48]. PLCs have 13 known isoforms that can be classified based on function and structure and they are PLC beta (β1–4), PLC delta (δ1,3 and 4), PLC epsilon (ε), PLC eta (η1–2), PLC gamma (γ1–2), and PLC zeta (ζ) [28,47,49,50,51,52]. PLC isoforms generally function as enzymes involved in protein kinase C activation via DAG and release Ca^2+^ from intracellular stores [28,46,47,48], all of which share a similar structure with greatly conserved catalytic X and Y domains which are responsible for PIP_2_ hydrolysis. PLCs also comprise EF-hands, which are the Ca^2+^ binding structures in the enzyme; a pleckstrin homology (PH) domain that is generally used for targeting the enzyme substrates; and a C2 domain, which is also essential in Ca^2+^ activity [53,54,55]. All PLC isoforms may function similarly, but they do differ in tissue distribution and regulatory mechanisms, and even have additional functions that make them variable from each other [55]. However, most investigated relevant PLCs were unable to elicit physiological patterns of Ca^2+^ release following microinjection into oocytes [56].

The specific PLC isozyme responsible for Ca^2+^ release at OA was first identified using mouse expressed sequence tag (EST) databases to describe a novel, testis-specific PLC, termed PLCzeta (PLCζ), a ~74 kDa protein in mice, its immunodepletion from sperm extracts suppressed Ca^2+^ release at OA [4,57]. Recombinant PLCζ injection in the form of protein or cRNA into mouse oocytes caused Ca^2+^ release similar to those at natural fertilisation [57,58,59]. The amount of PLCζ protein injected/expressed in mouse oocytes that resulted in successful Ca^2+^ release and OA corresponded to the same range as the amount of PLCζ found in a single sperm, estimated to be ~40 fg, which was also found to be the level at which PLCζ is most effective [32,44,57,58,59,60]. Therefore, PLCζ is the only protein that is shown to satisfy the requirements needed to be the sperm factor, as it is the only one that can induce Ca^2+^ oscillations which are seen during fertilisation [45].

The suggested PLCζ mechanism of action is that PLCζ targets the cytoplasmic vesicle-bound PIP_2_ in the oocyte, yielding IP_3,_ which targets the IP_3_R on Ca^2+^ stores, such as the endoplasmic receptors, to release intracellular Ca^2+^ [61,62,63]. RNA interference (RNAi) experiments targeting PLCζ in mice led to an early inhibition of Ca^2+^ release before OA, with such mice yielding a decreased number of offspring [57]. PLCζ, like other PLC isoforms, elicits Ca^2+^ release from intracellular stores via hydrolysation of PIP_2_ into DAG and IP_3_ [10,28,43,44,45]. However, given its high Ca^2+^ sensing ability and the distribution of the protein mainly in sperm and testes [55], PLCζ has currently been suggested to primarily function at fertilisation, inducing oocyte activation and embryogenesis [54,64,65,66].

## 9. PLCζ Structure and Function

**Figure 1:**
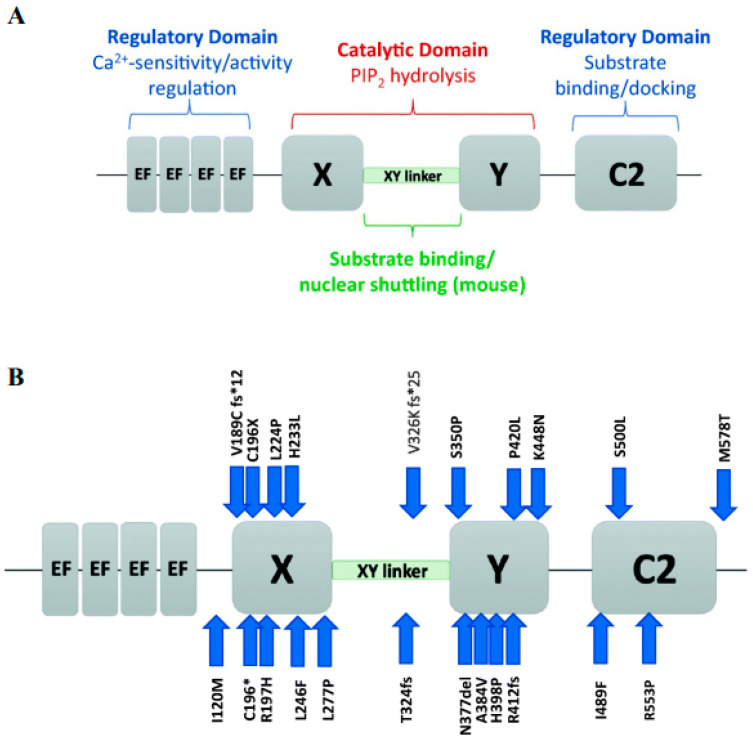
Schematic representation of PLCζ, briefly (**A**) summarizes the functional roles of the domains of PLCζ, including the regulatory EF-hands and C2 domains (blue text), and the X and Y catalytic domains (red text) connected by the X-Y linker (green text). (**B**) PLCζ functional domains, indicates the location of mutations identified in the literature, as indicated by blue arrows. Each mutation is represented by the original amino acid, followed by the amino acid position number, and then the mutated amino acid. Reprinted with permission from Ref. [55]. Copyright 2020 Journal of Assisted Reproduction and Genetics.

PLCζ is currently the smallest known PLC isoform (ranging in size from 70–75 kDa) [44,45,54,57], sharing a similar structure distribution as other PLC isoforms (Figure 1A), with an up to 60% similarity in its X and Y domains, especially with PLCδ1 [55]. The X and Y domains are said to consist of eight repeating units of beta/alpha helixes [44,67], where they play an essential role in fertilisation [68,69,70,71]. Moreover, the XY linker region, connecting the X and Y domains, exhibits significant species-dependant differences. Interestingly, the PLCζ does not have a PH domain [55], so the PLCζ targeting the membrane-bound substrate would have to be carried out by another mechanism, such as through the XY linker and possibly the C2 domain [55]. Removal of the C2 domain of PLCζ resulted in only a slight decrease in Ca^2+^ sensitivity and binding [53], suggesting that the C2 domain is not involved in Ca^2+^ sensitivity but rather Ca^2+^ oscillatory activity [45,54]. The C2 domain can also interact with phospholipids, such as PI(3)P and PI(5)P, indicating the possibility of the C2 domain being associated with targeting these phospholipids [54,67,72]. This notion was supported following the identification of homozygous PLCζ mutations in two infertile patients, which, while theydid not necessarily affect enzymatic activity in vitro, the mutated PLCζ exhibited a significantly lower affinity in binding to PI(3)P and PI(5)P [45,54,73].

PLCζ is also involved in nuclear sequestration activity that directs the protein to act in a cell cycle-dependent manner [28,74,75]. Inhibiting pronuclear formation resulted in persistent Ca^2+^ oscillations for an extended period of time [76,77]. This nuclear sequestration is attributed to a specific ‘nuclear localisation’ sequence found in the XY linker region of at least mouse PLCζ. Indeed, the presence of accumulated tagged-PLCζ in nascent pronuclei correlated with pronuclear formation [28,78,79,80], while the release of this tagged-PLCζ back into the cytoplasm corresponded to the pronuclear breakdown before mitosis, coinciding with the resumption of Ca^2+^ release [28,78]. The EF-hand region in PLCζ consists of four EF-hand motifs, each structured into a helix-loop-helix confirmation located at the N terminal of the protein. The EF-hand region not only plays a crucial role in Ca^2+^ sensitivity, distinguishing it from other PLC isoforms, but also perhaps exerts a role in nuclear translocation during fertilisation and binding to PIP_2_ [4,44,79,81]. Interestingly, the truncation of three out of four EF-hands led to an accumulation of PLCζ in the pronuclei [81]. PLCζ, through its EF-hands, exhibits supreme Ca^2+^ sensitivity [53], allowing it to be active even at basal oocyte cytosolic Ca^2+^ levels after gamete fusion [45,53]. Truncation of the EF-hands, or replacement with another PLC isoform EF-hands altered the Ca^2+^ sensitivity of the altered PLCζ but did not affect the enzymatic function [45,53,72]. Apart from a shared nuclear translocation role with the XY linker, the EF-hands may also play a further shared role with the XY linker in residue binding due to the presence of basic residues [53,54], illustrated by a decreased PIP_2_ interaction following the deletion of EF-hands [4,45,67].

The duration and frequency of PLCζ-induced Ca^2+^ oscillations are also an important part of fertilisation, which varies between species, extending from minutes to hours in terms of duration [43,63,82,83]. The precise amount of PLCζ is what determines the number of oscillations that can be induced during fertilisation. Indeed, increasing the amount of PLCζ injected into human oocytes resulted in an elevation in Ca^2+^ oscillation frequency and amplitude [54,84], this then can affect the level of gene expression found in the oocyte [23,54,85]. The amount of PLCζ needed to activate the oocyte also seems to differ between species [28,75,86]. PLCζ is currently understood to localise in the oocyte cytoplasm, specifically within intracellular vesicles [79,81,87,88]. Indeed, most PIP_2_ hydrolysation occurred in the cytoplasm, corresponding to PLCζ localisation in the cytoplasm near the nuclear envelope rather than the plasma membrane [69,89]. Furthermore, oocyte cell membranes do not exhibit any discernible PLCζ localisation, while depletion of plasma membrane PIP_2_ did not significantly affect Ca^2+^ oscillations at fertilisation [90]. Interestingly, the fusion of inositol lipid phosphatase with inactive PLCζ and injection into mouse oocytes to diminish PIP_2_ in vesicles led to the inhibition of Ca^2+^ oscillations [90]. This collectively suggested that PIP_2_ hydrolysis from intracellular vesicles, rather than the plasma membrane, is the primary source of the cytosolic Ca^2+^ oscillations in oocytes induced by PLCζ [89].

Intriguingly, transgenic mice where PLCζ was knocked out (KO) in two different studies indicated that KO male mice were able to have offspring but with a remarkably decreased amount than usual following in vitro fertilisation (IVF) alongside abnormal and delayed Ca^2+^ oscillations and an increased amount of polyspermy. However, ICSI of such KO sperm was unable to successfully elicit Ca^2+^ release [54,64,65,91]. Collectively, both studies indicated that although PLCζ plays an indispensable role at OA, it is possible that further factors may contribute towards Ca^2+^ release at fertilisation, in addition to (and perhaps independently of) PLCζ (discussed later in this review).

## 10. Abnormal Expression and Localization in Sperm

PLCζ has been found in the sperm of many different species and it typically localizes to different subcellular areas of the sperm head. For instance, it has been shown that PLCζ in mice is localized in the sperm’s head post-acrosomal region [92]. However, this pattern interestingly changes during capacitation [92]. However, in unincapacitated human sperm, PLCζ is mainly located in the sperm’s head, specifically in the equatorial region [93,94]. Another study showed that PLCζ in species, such as hamsters and mice, it is localized in the sperm’s head/acrosomal region [94]. In porcine and mouse sperm, PLCζ has been found in the acrosomal and post-acrosomal regions, and PLCζ has also been noticed in porcine sperm tails [62,95,96]. As for equine sperm, PLCζ was discovered in the equatorial section, acrosome, and head, mid-peace [97]. It is still unknown, though, as to whether these populations are physiologically reliable. Numerous studies have found diverse patterns of PLCζ among the same mammalian species, frequently using the same antibody probe, which raises questions about specific PLCζ localisation [45].

Recent efforts, using specific antibodies and optimised protocols, specific patterns of PLCζ localisation in human sperm were identified including equatorial, equatorial + acrosomal, and a uniformly dispersed pattern, with a further pattern in the tail and the mid-peace of the sperm [98]. The equatorial region is where PLCζ is most frequently found in human sperm [43]. This is rational from a biological perspective, enabling the PLCζ release into the cytoplasm immediately after gamete fusion [43]. Indeed, studies show that the specific localisation of PLCζ was related to fertilisation success, with the acrosomal + equatorial pattern corresponding to a higher chance of successful fertilisation, while dispersed PLCζ in sperm had a lower capacity for fertilisation [98].

While PLCζ has been found to be localized at the sperm tail, it is still unclear whether these results are accurate [99]. However, a previous study used an equine sperm tail injection to induce Ca^2+^ responses in oocytes [97]. Therefore, the possibility that tail PLCζ may function either as an activator or facilitator in subsequent processes cannot be ruled out. However, according to a different study, the localisation of PLCζ in the sperm tail is just an artifact, because the researchers had concluded that antibody specificity is still a significant issue and that is why we must ignore the PLCζ1 localisation in the tail of the sperm [98]. The potential role of PLCζ1 populations in the sperm tail has not yet been investigated; more study is required to specifically address this possibility. Studies on other species additionally indicate that the capacitation process in sperm is also important in activating PLCζ. It was suggested that the protein is activated during capacitation through tyrosine phosphorylation, and interaction with Na/K ATPase α4 (ATP1A), epidermal growth factor receptor (EGFR) [63]. Henceforth, PLCζ plays a critical role in fertilisation, where any abnormality associated with the protein can lead to infertility.

An interesting assertion was made by Aarabi et al. [100], who suggested that PLCζ may be expressed by the epithelial cells of the epididymis, secreted in exosomes, which was then surface-associated with sperm. This could be a potential and novel aspect of understanding PLCζ expression. However, in addition to this specific study using these points to suggest that PLCζ was not the sperm factor in favour of the group’s own candidate (PAWP), this particular study is viewed with significant caution given that the antibodies used were notorious for non-specificity and was indicated as such by the authors themselves in their study. Very little validation was performed of such assertions, and there is also little consensus to support the authors claims that PAWP instead of PLCζ is the mammalian sperm factor given the specific physiological requirements for gamete function [101]. Thus, while potentially providing an explanation for tail and other localisations of PLCζ, much more work is required before any assertions can be made with certainty.

## 11. PLCzeta in Human Male Infertility

Similar to defective PLCs and abnormal Ca^2+^ signalling (and involved downstream pathways) in clinical conditions [102,103], defects in PLCζ have strongly been associated with specific cases of male infertility wherein OA or fertilisation is defective (OA-deficient; OAD). Generally, infertile males whose sperm fail to fertilise oocytes tend to exhibit abnormal expression of PLCζ in the sperm [98]. The higher the levels of PLCζ, the more likely fertilisation succeeds. Moreover, when a depleted PLCζ from sperm was used to fertilise a mouse oocyte, Ca^2+^ release was reduced. This shows that defects or absence of PLCζ may lead to the failure of fertilisation [7]. Indeed, a specific PLCζ quantity is needed for successful OA, which differs between species, and reductions in such amounts may result in defective OA/fertilisation [43]. Sperm from oligoasthenoteratozoospermic, teratozoospermic, and asthenoteratozoospermic patients have been found to have lower levels of PLCζ [56]. Furthermore, sperm from globozoospermic patients usually exhibit a low rate of success in OA [104], either due to a lack of PLCζ, or if present at reduced amounts, they exhibit an abnormal localisation pattern [56,61,105,106].

PLCζ levels may also be associated with specific sperm structures, as globozoospermic sperm with acrosomal buds selected from a population of sperm exhibiting a complete round-headed globozoospermic morphology could be used to achieve successful fertilisation without fertility treatment, also corresponding to an acrosomal pattern of PLCζ localisation [107]. Moreover, sperm from several patients exhibiting either absent or severely reduced levels of PLCζ were unable to induce Ca^2+^ release following injection into mouse oocytes [93]. However, when such sperm were co-injected with PLCζ mRNA in mouse oocytes, Ca^2+^ oscillations were rescued and OA/fertilisation was able to proceed [93]. Infertile, OAD males also tend to exhibit mutations in the PLCζ gene [68,69,108]. Indeed, injection of mutant PLCζ cRNA into mouse oocytes did not lead to sufficient patterns of Ca^2+^ release, resulting in failed OA in mouse oocytes, in stark comparison with oocytes injected with wild type PLCζ cRNA [68]. Numerous such mutations have now been identified by multiple independent studies and correlated with OA failure in humans (Figure 1B) [99,108].

## 12. Assisted Oocyte Activation (AOA)

AOA is a potential treatment for male-related infertility that aims to mimic physiological Ca^2+^ release [109,110]. AOA methods currently comprise of various modalities, consisting of either individual or combinations of electrical, chemical, and mechanical stimuli to activate oocytes during assessed reproductive technology (ART) methods, including IVF and ICSI [7]. AOA will produce either multiple or single Ca^2+^ oscillations. Single Ca^2+^ oscillations in some forms of AOA are not ideal for future successful development in humans and mice [109,110]

## 13. Electrical Activation

The electrical method has been tested in bovine and human oocytes [111], aiming to apply nanoscale electrostimulation on oocytes, allowing for an influx of extracellular Ca^2+^ through migration of lipid bilayer-charged proteins and pore formation within the membrane [112]. This results in a long duration of single rapid Ca^2+^ increase in the oocyte [111,113,114,115]. The success of this technique depends on the size of the pore formed and the extracellular Ca^2+^ concentration. However, the downside of such a method is the formation of excess reactive oxygen species (ROS), in addition to physical damage to the oocyte [116]. Interestingly, perhaps measuring the electrical resistance in a cell could also serve as a tool to detect oocyte viability and penetration [7], and thus while electrical AOA may not be an ideal clinical therapeutic, perhaps some modifications could yield a potential diagnostic of OA.

## 14. Mechanical Activation

Mechanical activation is the result of a mechanical disruption of the oocyte, resulting in a ‘manual’ release of Ca^2+^ via intracellular store disruption or manual elevations of Ca^2+^. This could be accomplished by piercing the oocyte, leading to increased Ca^2+^ influx, or direct microinjection of Ca^2+^ into the oocyte. Perhaps more invasively, another mechanism involves a physical ER membrane disruption and mitochondrial redistribution, or (more popularly) manual oocyte membrane disruption followed by vigorous oocyte cytoplasm disruption to increase the Ca^2+^ load. While of course significantly physically disruptive, such mechanisms would perhaps enhance closer contacts between sperm and intracellular membranes, further enhancing the chances of successful OA [7,117,118].

## 15. Chemical Activation

Chemical methods of activation stimuli utilise lipid-soluble chemicals termed ‘Ca^2+^ ionophores’ that diffuse into the oocyte and enhance Ca^2+^ permeability, Ca^2+^ influx, and release of intracellular stored Ca^2+^ [7]. Such ionophores include ionomycin, A23187 (calcimycin) [7,119], and ethanol [7] which all cause a single rise in Ca^2+^ [7,119]. There are, however, further agents that facilitate to multiple Ca^2+^ transients, which include thimerosal, phorbol esters, or strontium chloride (SrCl_2_) [7,119]. SrCl_2_ efficacy in human oocytes is still debatable [7]. Ionomycin and A23187 (calcimycin) are the main used agents in IVF for AOA. Thiomersal is not widely used because it causes oxidation of tubulin that will interfere with polymerization and spindle formation, thus is prevented by follow-up treatment with dithiothreitol. Calcimycin is an antibiotic that chelates Ca^2+^ and transports them through biological membranes. Ionomycin has a similar action but is more potent and is specific to Ca^2+^, and it stimulates gene expressions [7].

It is more effective to deliver Ca^2+^ ionophores after ICSI and not with it. Patients’ characteristics also play role in determining the success of ICSI and Ca^2+^ ionophores. Indeed, the effect in humans is not consistent; with some studies and meta-analyses indicating that the effect of Ca^2+^ ionophores in the case of sperm morphological abnormalities is negative, while other studies indicate positive results in cases with <30% successful fertilisation rates in previous ICSI cycles [7]. Further to such conflicting data, the success of AOA protocols is also determined by the concentration and length of exposure, the number of exposures, and the timing of exposure following ICSI, all of which play a role in activation success. Indeed, the literature exhibits heterogeneity in methodology success, making the broad application and evaluation of safety difficult, particularly since ionophores could be toxic to oocytes if the right parameters are not followed [7].

Some cases of successful OA after ICSI have been reported. However, ionophore treatment may hold cytotoxic, teratogenic, or even mutagenic effects for the embryo. For instance, the abnormal calcium-induced signal may have poor outcomes on epigenetic processes. Furthermore, current protocols may not be effective for all patients receiving this treatment [120]. The traditional concern of AOA use has always been that Ca^2+^ release following AOA methods differs from physiological release, specifically in the frequency and amplitude of Ca^2+^ release [56]. However, the application of AOA with ICSI did not affect embryo quality [121,122,123,124], and increased fertilisation rates [7]. Indeed, the application of AOA accelerated embryogenic cell division rates [125], and did not yield an increase in birth defects, rates of medical abortions, or congenital malformations compared to normal pregnancies. However, other studies again suggested that the application of A23187 specifically led to embryo degradation and to the failure of second body formation, [121,122,126,127,128,129]. There is a chance that the use of AOA will not avoid activation deficiency even with the use of ionophores [7], particularly if the problem is not entirely sperm-related [130].

Interestingly, however, chromosomal abnormality and defective embryogenesis following AOA could be overcome by supplementation of AOA media with granulocyte-macrophage colony-stimulating factor (GM-CSF), a cytokine involved in human preimplantation embryo development [129]. Indeed, such supplementation is in line with several studies that indicate that the supplementation of AOA protocols enhances successful OA and subsequent embryogenesis. Other chemical agents also include protein synthesis or protein kinase inhibitors, such as puromycin and 6-dimethylaminopurine (6-DMAP), respectively, which are most effective when used in combination with ionophores [7]. Indeed, such concurrent treatments are standard practice for AOA in domestic animals and are commonly used for OA after nuclear transfer. The reason underlying this need for multiple stimuli is dependent upon cyclin B synthesis, which is continuously present and stimulates CDK1 activity, and thus the meiotic arrest of mammalian oocytes [66,131].

A single Ca^2+^ transient would result in cyclin B degradation and reduction of CDK1 activity, promoting meiotic resumption [132], which may underly some of the success of single-transient AOA protocols in the clinic. However, a single Ca^2+^ transient would only result in a temporary alleviation of arrest, with cyclin B resynthesis followed by the resumption of CDK1 activity and re-arrest of the oocyte cell cycle [66]. To this degree, it would be perhaps advantageous to concurrently inhibit cyclin B synthesis with the termination of CDK1 activity via the prevention of protein kinase activity, or indeed eve inhibition of protein synthesis. This could perhaps explain why AOA is most effective with ionophore treatments when agents, such as puromycin/6-DMAP (protein kinase/protein synthesis inhibitors) are used [66,131,132,133,134]. Indeed, Ca^2+^ ionophore treatments seem more effective upon in vitro-aged oocytes following ovulation, perhaps due to a decline in cyclin B levels [66,131,132,133,134], and is perhaps an area requiring urgent investigation. Indeed, Tsai et al. [135] recently demonstrated that AOA application in older patients with a diminished ovarian reserve seemed to improve the resultant embryo quality, particularly in women aged ≥ 40 years.

## 16. Therapeutic and Diagnostic Options for PLCζ

In recent years, numerous studies have successfully demonstrated the induction of Ca^2+^ oscillations following injection of recombinant PLCζ RNA or protein, with both modes leading to successful OA and subsequent embryogenesis to the blastocyst stage at rates comparable to those achieved by IVF [110,120].

To this degree, production of active and pure recombinant PLCζ protein is another option for rescuing OA in case of ICSI failure, and other similar male infertility conditions [119]. The use of recombinant PLC holds the advantage of knowing the dose needed for administration from human assays in sperm (50–100 fg\sperm) [7]. However, a disadvantage to this method is the chance of over-injecting PLCζ, which can lead to the abnormal frequency and amplitude of Ca^2+^ oscillations and a low rate of blastocyst development [119]. Indeed, this is specifically detrimental to the utilisation of PLCζ RNA due to the potential for uncontrolled expression of PLCζ RNA in oocytes, even if it was successful in causing prolonged sperm-like repetitive transient Ca^2+^ waves. Other disadvantages with RNA injections is the chance that the dose of injected RNA is small or not enough for translation into PLCζ needed for Ca^2+^ influx or release from ER, or it may lead to abnormal Ca^2+^ release [7] following a delay by 15–20 s compared to PLCζ protein utilisation [136]. Surprisingly lower doses of PLCζ RNA injection were more effective than higher doses. Using these parameters to compare PLCζ mRNA used with other methods, including cytosolic aspiration, electrical stimulation, and ionomycin treatment, PLCζ RNA utilisation is a better therapeutic agent. However, the ultimate decision for the applicability of PLCζ mRNA as a therapeutic agent needs a further trial with the treatment leading to the full-term development of the embryo with no side effects. Another disadvantage to this method is that the protein is continuously expressed, making it difficult to control the frequency of Ca^2+^ oscillations, which is important in proper embryogenesis. Furthermore, the average half-life of mRNA molecules is 9 h, making it difficult to exist in cells beyond that time. Further, mRNA lacks the ability to integrate into the host genome, thus generating induced pluripotent stem (iPS) cells [84].

**Figure 2:**
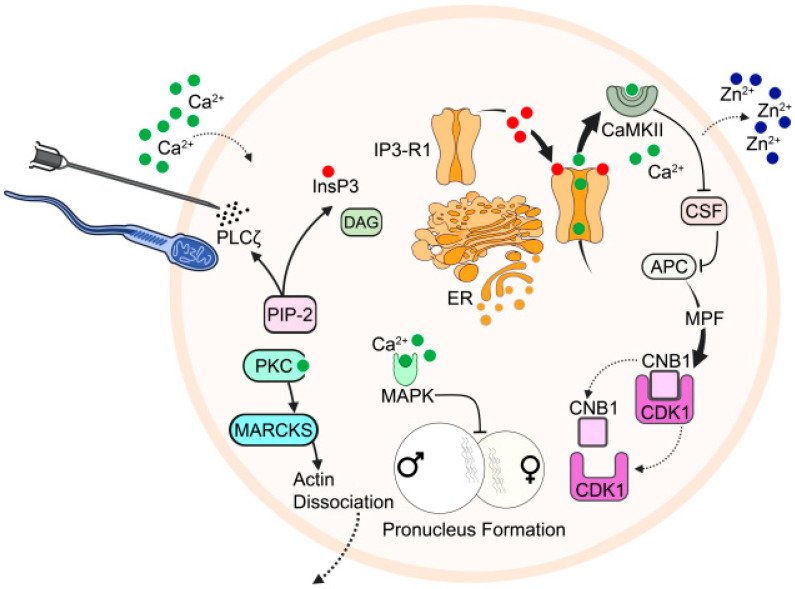
Schematic representation of the mechanistic function of PLCζ underlying Ca^2+^ release at fertilisation, with associated processes resulting from the completion of oocyte activation. The release of PLCζ from sperm, or even injection into the oocyte, hydrolyses PIP_2_, yielding DAG and IP_3_. IP_3_ binds to specific IP_3_R on the ER, triggering Ca^2+^ release, and Ca^2+^-induced-Ca^2+^-release (CICR). Released Ca^2+^ activates CaMKII, which phosphorylates EMI2 (CSF), releasing APC/C from its usual inhibition that otherwise maintains cell cycle arrest. Ca^2+^ release is also linked to the release of Zn^2+^ at the Zn^2+^ spark, which also down-regulates EMI2 due to a decrease in intracellular Zn^2+^ availability. Active APC/C further causes ubiquitination of cyclin B1, resulting in inactivation of MPF, releasing MII arrest. Concurrently, Ca^2+^ also activates protein kinase C (PKC), which phosphorylates myristoylated alanine-rich C kinase substrate (MARCKS), which disassociates from F-actin, causing actin breakdown in the oocyte cortex, allowing for cortical granule exocytosis. Ca^2+^ release also inactivates mitogen-activated protein kinase (MAPK), leading to pronuclear formation. Figure is an original work, but inspired by [155].

Thus, while the utility of recombinant PLCζ represents a potential therapeutic option for OAD patients, perhaps even for a wider range of patients where fertilisation occurs, but embryogenesis is poor [56], reliably generating purified recombinant PLCζ remains to be established, with further focused clinical trials required to ascertain applicability. Furthermore, administration of recombinant therapeutic PLCζ (either RNA or protein) currently requires co-injection with sperm, which is not entirely accurate in terms of delivery of specific amounts of PLCζ. Considering that the amount of PLCζ protein delivered to the oocyte directly underlies embryogenic quality, current injection methods may not necessarily enhance current success rates achieved with AOA [7,56]. As such, while the therapeutic application of recombinant PLCζ is exciting and represents a potentially very powerful clinical tool, much more work is required before clinical application (Figure 2).

Considering the large body of work examining PLCζ levels and localisation patterns in mammalian, and particularly human, sperm, it is prudent to consider PLCζ as a powerful diagnostic indicator of sperm fertility not just in cases of OAD, but also perhaps a larger range of male factor conditions and cases of poor/abortive embryogenesis [56,110]. Indeed, recent studies have correlated specific localisation patterns and levels of PLCζ with successful fertilisation and parameters of sperm fertility [98]. However, while sperm PLCζ has been correlated with sperm DNA fragmentation [137,138,139], abnormal sperm parameters and morphology [98,140,141,142,143,144,145,146,147,148,149,150,151,152,153], abnormal embryogenesis [154], and perhaps also a potential utility for round spermatid injection [98], most such analyses have assessed sperm PLCζ RNA rather than protein [98]. Given that the role of not just PLCζ RNA but all sperm RNA at fertilisation is considered limited, much more work is required to establish links between PLCζ protein and the sperm defects examined with specific tools and established protocols [98]. An indirect approach to examine sperm PLCζ deficiency has employed the use of human sperm microinjection into mouse oocytes and examination of the resultant Ca^2+^ release, known as the mouse oocyte activation test (MOAT). However, given that human PLCζ is significantly more potent in activity compared with mouse PLCζ in mouse oocytes, even minimal human PLCζ could result in high frequency and amplitude Ca^2+^ oscillations, and would thus perhaps not represent a method to examine cases of reduced PLCζ, but cases of outright absence [98].

**Figure 3:**
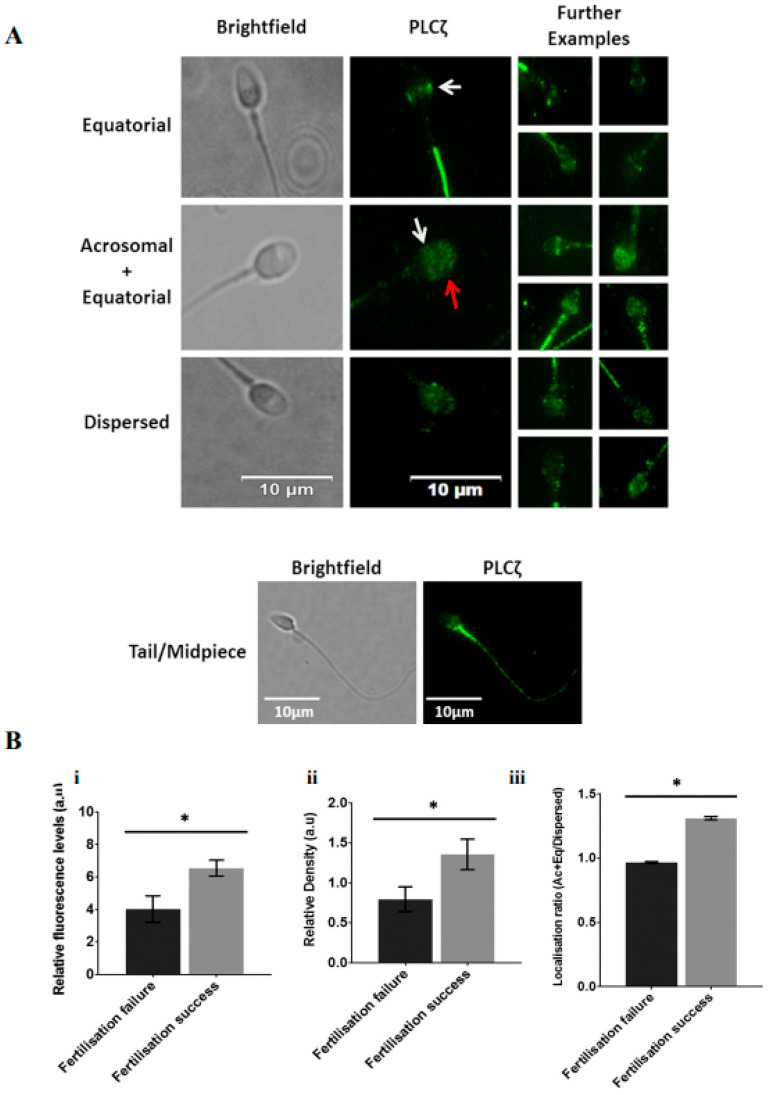
(**A**) Representative immunofluorescence images of observed PLCζ localisation patterns in human spermatozoa. Brightfield (leftmost panels) and PLCζ (green fluorescence; middle panel) images were obtained. Predominant localisation patterns observed include equatorial (white arrows), acrosomal + equatorial localisation (red arrow indicates acrosomal localisation), and dispersed localisation patterns. The leftmost panels include further examples of each pattern, illustrating the lack of uniformity of patterns. The bottom-most panel indicates fluorescence observed in the mid-peace and tail. White scale bars represent 10 μm. (**B**) Histograms indicating differences in (**i**) relative fluorescence, (**ii**) relative density and (**iii**) Ac + Eq/dispersed localisation ratio of sperm PLCζ between cases of fertilisation failure and fertilisation success following fertility treatment. Asterisks (*) indicate statistically significant differences (*p* ≤ 0.05). Data indicate the potential diagnostic capability of sperm PLCζ parameters in indicating potential fertilisation success. Reprinted with permission from Ref. [99]. Copyright 2020 Andrology.

Thus far, sperm PLCζ protein has predominantly been examined using immunocytological analyses [94,98,152,156,157]. Indeed, while current ART clinics possess at least basic microscopy facilities conferring the capability to perform such methods, the main issue lies with antibody and methodology variance and specificity, with most studies relying upon antibodies (predominantly only one) with low PLCζ specificity. We can quantify PLCζ protein levels through immunofluorescent staining with an anti-PLCζ antibody and compare the relative fluorescence of the PLCζ levels in the sperm (Figure 3). Furthermore, Kashir et al. [157] concluded that while OAD sperm exhibited lower immunofluorescence for PLCζ compared to normal subjects, a high variability in the immunofluorescence levels of both patients and controls was noted, where some control patients had immunofluorescence levels similar to OAD patients. Since mouse oocytes require 20–50 fg PLCζ to undergo activation, a similar statement with unknown ranges can be said about human oocytes, inconsistent or inaccurate methodology may result in misdiagnosis [158].

## 17. Alternative Diagnostic and Therapeutic Targets of OA

Given the considerable amount of data present in the literature pertaining to PLCζ, the importance of this enzyme is apparent for potential therapeutic/diagnostic applications. However, several issues remain regarding its clinical utilisation, related to both technical aspects, but also perhaps pertaining to the incomplete picture regarding the role of PLCζ in OA. Indeed, the independent PLCζ KO studies, while supporting the importance of PLCζ at OA, also suggest that alternative contributory mechanisms may also be present [54,64,65,91]. Indeed, it is possible that an alternative ‘cryptic’ sperm factor(s) may be present within sperm, which may facilitate or complement PLCζ action [159]. While any clues regarding the absence/presence of such a cryptic factor remains to be elucidated, several molecular players are involved during the complex series of concurrent events known as OA.

## 18. Actin-Mediated Cytoskeletal Movements

A particular example of this is actin, perhaps the most conserved and abundant family of proteins in eukaryotic cells, may possess specific roles in the oocyte cortex development and fertilisation [160]. Indeed, actin exhibits high-affinity binding to Ca^2+^, suggesting that actin could act as an intracellular buffer to store and release [161,162,163]. Based on this, using latrunculin A (LAT-A) and mycalolide B, which are actin-depolymerizing agents, on the mature egg of *A. aranciacus* at the optimum period of fertilisation, induced an increase in Ca^2+^ and depolarization of plasma membrane after activation [164,165,166]. New evidence provided by recent studies supports the importance of actin in controlling the events of oocyte maturation, OA, and cleavage. Comparing the organization and morphology of cortical actin cytoskeleton in immature and mature oocytes provide a better understanding of the cortical F-actin structure role in regulating normal egg maturation and monospermic fertilisation [167].

In *Drosophila,* actin was found to be smoothly distributed before OA, the onset of which resulted in actin spreading out, with a relaxed actin cytoskeleton required for initiation and propagation of Ca^2+^ release, which in turn leads to a reorganization of actin in a wavelike manner [168]. Drugs promoting F-actin depolymerization or stabilization on the fertilisation reaction of sea urchin eggs resulted in the modification of the actin structure and dynamics, which in turn altered Ca^2+^ release patterns [169]. Following fertilisation, the actin cytoskeleton visibly reorganizes at the point of gamete fusion. Interestingly, actin bundle formation requires an elevation of Ca^2+^ levels, while detachment and cortical translocation of actin is a prerequisite for normal cellular cleavage, indicating an important role for Ca^2+^-dependent actin reorganisation [167,170,171,172]. It was suggested that heparin- or age-induced hyperpolymerization of the starfish egg cortical actin disrupted cytoskeletal dynamics at fertilisation, which in turn detrimentally influenced Ca^2+^ release [167,173,174,175]

Considering that the relationship between actin and Ca^2+^ could be viewed as one where actin acts as a buffer to store and release Ca^2+^ [161,162,163], it is thus possible that such a phenomenon could be affecting the timing of cellular cleavage apart from other events in cell division, such as cleavage furrow formation, nuclear envelope breakdown, and reformation [176]. To this degree, particle image velocimetry (PIV) detected specific rhythmic cytoplasmic movements due to contraction of the actomyosin cytoskeleton triggered by Ca^2+^ oscillations. This is a non-invasive and safe diagnostic method and can also be related to the development potential of forming zygotes. This test can be used after the injection of PLCζ cRNA into the human oocyte. PIV was used in humans for imaging post-microinjecting with PLCζ cRNA in oocytes that failed ICSI. These oocytes were donated by patients and microinjected with PLCζ cRNA with a mixture of substances using a micropipette needle with a brief electrical pulse. The first Ca^2+^ spike was delayed by 15–20 s with the use of PLCζ cRNA compared to normal sperm injection. This correlates with the translation of PLCζ protein. The cytoplasmic movement follows Ca^2+^ oscillation pattern, the higher the Ca^2+^ peak, the slower the movement [136,177]. This movement depends on the actin cytoskeleton and is influenced by the presence of the sperm. This was proven by the failure of oocytes injected with PLCζ cRNA without prior ICSI to show cytoplasmic movement [136]. In summary, the PIV can be used to decide on the success of inducing Ca^2+^ oscillations by confirming cytoplasmic movement, which could be used as a diagnostic predictor of OA efficacy and thus embryogenesis [177].

## 19. Modulators of Ca^2+^ Homeostasis

Store-operated calcium entry (SOCE) is a system that maintains Ca^2+^ cytosolic concentration when ER stores are depleted. The major components of the SOCE are sarco-ER Ca^2+^-ATPase (SERCA), Ca^2+^ release-activated Ca^2+^ channel protein 1 (ORAI1), stromal interaction molecule-1 (STIM1), and other membrane channels. Targeting these proteins may produce Ca^2+^ oscillations without PLCζ. STIM1 senses Ca^2+^ stored in the ER, and with the help of a sterile alpha motif domain, STIM1 polymerizes to the plasma membrane yielding to the protein-protein interaction with ORAI, which will result in extracellular Ca^2+^ influx. Any mutation in STIM1 leads to a persistent influx of Ca^2+^ regardless of ER status. CaMKII and mitogen-activated protein kinase (MAPK) are proteins responsible for progression in MII and pronuclei formation, any modulation in their function can affect OA, making them a potential therapeutic option. These systems are believed to have a role in spontaneous oocyte activation (SOA) [152].

SOA is a phenomenon where the oocyte decides to exit MII, enter anaphase II and form a single pronucleus without any interaction with sperm. This could perhaps be explained by changes in cell cycle regulators, post-ovulatory oocyte aging, and temperature changes during oocyte harvesting. Such a concept is the extreme opposite to infertility resulting from failure of sperm to activate oocytes through PLCζ and Ca^2+^. One proposed mechanism is the elevation of LH which can initiate Ca^2+^ release. However, oocyte collection without any hormonal stimulation also revealed SOA, excluding LH as a possible cause. Another theory explains SOA due to problems in cell cycle regulators that arrest oocytes at MII, c-mos KO mice showing SOA can support this hypothesis. Some patients showed a repeated incidence of SOA highlighting the possibility of a genetic cause [178]. Perhaps some oocyte molecular factors that could explain SOA are STIM1 and ORAI1 at SOCE, or perhaps CAMKII/MAPK, which are Ca^2+^-ATPases or Ca^2+^-dependent proteins. MAPK early decrease in addition to activation of spindle assembly checkpoint proteins may have an input in SOA [178].

Further to such aspects, plasma membrane Ca^2+^ ATPase 1 (PMCA1) protein support Ca^2+^ efflux at fertilisation and the proper growth, weight, and body composition of the ensuing offspring, is indicated in mice oocytes. PMCA1, along with other proteins, such as SERCA2B, functions in decreasing cytoplasmic Ca^2+^ levels following each Ca^2+^ transient. Furthermore, two Ca^2+^ influx channels, TRPM7 and Cav3.2, increase cytosolic calcium [6]. TRPM7 senses the extracellular concentration of Ca^2+^ and Mg^2+^ to control Ca^2+^ influx [7]. A lack of these causes subfertility, since threshold calcium levels are not attained [6] and lead to the premature end of Ca^2+^ oscillations [7]. Obesity and inflammation also impact physiologic calcium oscillations through their effect on the redox balance and mitochondrial function [6]. Modulating mediators that control Ca^2+^ influx, such as TRPM7 and CaV3.2, can maintain Ca^2+^ oscillations [7]. In starfish, gamete fusion activates a voltage-gated Ca^2+^ channel [179,180], while both voltage-gated channels and NAADP underlie Ca^2+^ release in sea urchins [181]. While IP_3_-dependent Ca^2+^ release is an essential component of OA for at least mammalian species, others utilise alternative or additional pathways [26]. For example, cADPR can also induce Ca^2+^ release via perhaps the ryanodine receptor in sea urchin fertilisation [182,183], while evidence also exists for a role of NAADP in sea urchins and starfish [184,185,186]. Some species, such as *Drosophila*, induce OA before gamete fusion, mediated via extracellular Ca^2+^ in response to a physical compression of egg plasma membrane TRP channels during ovulation [187,188] (although the propagation of the Ca^2+^ is still IP3 receptor-mediated [188]).

In other species, Ca^2+^ influx supplements cytoplasmic Ca^2+^ release at OA in echinoderms, molluscs, and worms [26]. Other such species include zebrafish and *Sicyonia* shrimp, which involve an extracellular induction of Ca^2+^ without sperm involvement [189,190], presumably due to extracellular ionic concentrations. Indeed, shrimp egg Ca^2+^ waves seem initiated by magnesium ions (Mg^2+^) in the extracellular milieu [26,190]. As previously discussed, TRPM7 senses extracellular Ca^2+^ and Mg^2+^ to control Ca^2+^ influx [7]. Indeed, the ratio of Mg^2+^:Ca^2+^ in culture media may exert a role in AOA, as decreasing the Ca^2+^:Mg^2+^ ratio increased Ca^2+^ release within the oocyte [7]. Indeed, extracellular factors may yet be playing a significant (yet under-appreciated) role in determining the success of OA. Changes in salinity and pH affect the OA and fertilisation in sea urchins, with both dilution and acidification of seawater exerting significant detrimental effect upon the efficacy of OA and fertilisation [191]. Furthermore, in addition to the external physical stimuli required for *Drosophila* egg activation, osmotic pressure generated by the uptake of external fluid drives the initiation of Ca^2+^ release. This mechanism is regulated by conserved osmoregulatory channels, aquaporins, and DEGenerin/Epithelial Na^+^ channels, utilising transient receptor potential M channels to transport Ca^2+^ across the plasma membrane into the egg [192].

## 20. The Role of Zinc (Zn^2+^)

Perhaps the most intriguing non-Ca^2+^ related to OA are the intracellular levels of Zn^2+^, levels of which increase before fertilisation, while after fertilisation, Zn^2+^ levels decrease, correlating to the release of meiotic arrest [109]. The chelation of Zn^2+^ leads to cell cycle promotion in oocytes, whilst also regulating the function of CDC25, which in turn regulates maturation-promoting factor (MPF) [178,193], early mitotic inhibitor 2 (EMI2) [178,193,194], and zinc-binding domain in CSF (i.e., the molecular players involved in maintenance of oocyte MII arrest). Indeed, multiple techniques have utilised this dependency for AOA protocols using Zn^2+^ chelators to trigger a resumption of MII in human oocytes. This concept can be used to treat fertility due to the failure of OA with Ca^2+^ [178,193]. A Zn^2+^ chelating agent, N,N,N′,N′-tetrakis(2-pyridinylmethyl)-1,2-ethanediamine (TPEN), enabled the effective completion of MII and blastocyst development in pigs, but to a lower extent compared to other Ca^2+^ ionophores [7]. The absence of intracellular Zn^2+^ with heavy metals led to the activation of the oocyte and miotic resumption without changes in Ca^2+^ levels. Indeed, TPEN affects Zn^2+^ levels without altering intracellular Ca^2+^. In mice, TPEN resulted in blastocysts with lower inner cell mass and trophectoderm cell quantity. The effect of TPEN use in humans is not well established and does not seem entirely effective [109].

Imaging experiments indicated that mouse OA triggered transient ejection of Zn^2+^ into the extracellular milieu in a series of events called the ‘zinc spark’ [178,195,196], immediately following the first Ca^2+^ transient. Similar observations have been made in human, bovine, porcine, and primate systems [197,198], suggesting (like Ca^2+^) that this Zn^2+^ spark is highly conserved (at least in mammals) [199,200]. Although recent studies do suggest that a similar process involving Zn^2+^ depletion at fertilisation occurs in *Drosophila* [201] and zebrafish [202]. Immature mouse oocytes are unable to elicit a Zn^2+^ spark, indicating Zn^2+^ accumulation is required during meiotic maturation [203,204]. Analysis of Zn^2+^ spark dynamics indicated that zygotes successfully able to reach the blastocyst stage released more Zn^2+^ compared to those unable to develop [205], suggesting that perhaps quantification of Zn^2+^ could represent a diagnostic marker of embryogenic capacity in mouse zygotes [206].

## 21. Conclusions

Given the complexities underlying OA at fertilisation, it is astounding that much work has been accomplished, establishing the mechanisms underlying Ca^2+^ release, the indispensable involvement of the sperm factor PLCζ, and the utilisation of both these players in a therapeutic and diagnostic context. However, research has yet again demonstrated that there is yet much more to be elucidated, particularly regarding the role of seemingly disparate, yet utterly interdependent actors, such as Zn^2+^, modulators of Ca^2+^ homeostasis, and the mechanisms of actin cytoskeleton dynamics. The discussion surrounding OA has traditionally revolved around the intracellular Ca^2+^ release and PLCζ (at least within mammals). While these aspects are without a doubt integral to the process, it is increasingly clear that just these by themselves do not constitute the ‘end sum game’ OA. Indeed, as our understanding of several intra- and extracellular aspects surrounding OA increases, it becomes clear that OA (including intracellular Ca^2+^ release and PLCζ) need to be viewed as part of a much larger, interconnected, and vastly more complex overview. Indeed, much promise is present for the therapeutic and diagnostic targeting of such players, although much more work is yet required to fulfil this potential.

## Data Availability

Data sharing not applicable.
